# Types of decorations, their social meaning and influence on moral injury: A mixed methods approach

**DOI:** 10.1371/journal.pone.0333344

**Published:** 2025-10-27

**Authors:** Ruud Lathouwers, Tine Molendijk

**Affiliations:** 1 Department of Social Psychology, and Academic Collaborative Center for an Inclusive Labor Market, Tilburg University, Tilburg, Netherlands; 2 Faculty of Military Sciences, Netherlands Defense Academy, Breda, and Radboud University, Nijmegen, Netherlands; Xiamen University Malaysia, MALAYSIA

## Abstract

Research on moral injury has thus far paid little attention to social factors and cultural elements. This study addresses this gap by examining the effects of one particular cultural artefact on moral injury that is typical of armed forces, (military) decorations, including awards and medals. Through a preregistered experiment involving two scenarios—omission of helping behavior and commission of mortar fire—resulting in civilian deaths, we manipulated the factors self-attribution versus attribution of blame to others/the system and the presence or absence of a decoration. Moreover, we conducted seven qualitative interviews. Contrary to our expectations, none of the experimental conditions within the scenarios yielded significant differences. However, the scenarios themselves differed significantly from each other, indicating that wrongful inaction may have a stronger effect on moral injury than wrongful action. The subsequent qualitative interviews (**N* *= 7) revealed nuanced insights, suggesting that the effects of decorations on moral injury may vary. Cases were discerned in which military decorations demonstrated the potential to either alleviate or exacerbate moral injury. This dynamic depended on factors such as an individual’s assessment of the justifiability of the act for which they received the decoration, and the perceived authenticity of the decoration’s bestowal – whether it was experienced as a genuine acknowledgment or merely a superficial gesture. These findings indicate a possibly complex interplay of cultural artifacts and moral injury within military contexts.

## Introduction

Jonathan Shay, the founding father of ‘moral injury’ (short defined as trauma and/or stress due to a moral transgression [1]), first introduced the concept in his seminal work Achilles in Vietnam [1]. In this book, he recounts numerous stories from soldiers. Shay opens his book with a situation in which a soldier was ordered to fire upon what were suspected to be enemy soldiers on boats unloading armaments, only to discover that they were civilians.

So you know in your heart it’s wrong, but at the time, here’s your superiors telling you that it was okay. So, I mean, that’s *okay* then, right? This is part of war. Y’know? Gung-HO! Y’know? “AirBORNE! AirBORNE! Let’s go!” So we packed up and moved out. They wanted to give us a fucking Unit Citation – them fucking maggots. A lot of medals came down from it. The lieutenants got medals, and I know the colonel got his fucking medal. And they would have award ceremonies, y’know, I’d be standing like a fucking jerk and they’d be handing out fucking medals for killing civilians. [1, p4]

The experience of this veteran is particularly compelling as it demonstrates the profound moral significance of military decorations. It underscores that both receiving and witnessing the awarding of decorations can hold deep moral meaning and be experienced in various ways. Research confirms that social context, including rituals and cultural artifacts, plays a crucial role in relation to moral injury [2]. While, of course, the socio-cultural context is broader than just decorations, they are clearly a notable aspect of it. Nevertheless, research on these factors is remarkably scarce. This is particularly surprising given that military decorations hold significant importance in military culture. From the image of the heroic World War Two veteran, with his Medal of Honor, such as seen in the movie Hacksaw Ridge, to the North Korean general plated with medals, decorations are a salient military tradition. Hence, the current research assumes the following research question: “Does receiving a decoration have an effect on moral injury, and if so, how?”

### Moral injury

A significant proportion of soldiers worldwide develop feelings of shame, guilt and/or betrayal and anger as a result of their deployment experience, with estimates ranging from approximately 5% to 25% [3–6]. The concept of moral injury was introduced to extend the understanding of trauma toward the inclusion of ethical and sociological perspectives. As mentioned, the concept of moral injury was introduced by Shay [1]. His – often-used – definition of moral injury is “(a) betrayal of what’s right, (b) by someone who holds legitimate authority (e.g., in the military – a leader), (c) in a high stakes situation” [1, p182]. A second much-cited description is Litz et al.’s “the lasting psychological, biological, spiritual, behavioral, and social impact of perpetrating, failing to prevent, or bearing witness to acts that transgress deeply held moral beliefs and expectations” [5, p697].

The two definitions can be seen as complementary, highlighting different possible aspects of moral injury. Whereas Litz et al.‘s [5] definition emphasizes self-blame resulting from acts of omission or commission by the individuals themselves, Shay’s definition focuses on the blaming of trusted others, or even “the system” at large (e.g., military organization, government) because of their acts of omission or commission [1,7–9]. Notably, soldiers may frequently experience blame directed both inwardly and outwardly simultaneously [10]. The trusted “others” on which outward blame is focused may include both the military organisation, the political leadership and broader society.

Today, the concept of moral injury has firmly entrenched itself in research on military trauma. Most contemporary studies on moral injury are dedicated to developing practical clinical models, facilitating clinical assessment and diagnosis, exploring its relationship with PTSD, and devising therapeutic interventions (see, for instance, the following reviews [7–9]). While crucial, this clinical emphasis has reproduced an intra-individual focus, overlooking the broader social context [11,12]. In most current studies, Shay’s work is often omitted. This study rekindles attention to moral injury’s social dimension.

### Decorations and the social context of moral injury

Military decorations serve a social function by demonstrating the acknowledgment and approval of the organization bestowing the decoration [13]. As Gavriely-Nuri notes, “the distribution of decorations is an effective symbolic act that promotes and justifies the use of military force” [14, p403]. In a more general phrasing, decorations signify in a tangible manner that the military organization acknowledges and supports the actions performed by the soldier in a specific mission.

The dimensions of moral injury outlined above underscore its fundamentally relational character, involving both institutional betrayal, when soldiers perceive that military or political authorities have failed them morally, and self-condemnation. On the other side of the medal lie recognition and self-worth. The concept of institutional betrayal reflects a profound breach of trust between the individual and the organization responsible for their welfare and moral guidance [15]. In contrast, institutional recognition, in which the organization openly validates soldiers’ experiences and sacrifices, can serve as a protective or reparative mechanism, mitigating the effects of moral injury [15]. This duality reflects broader social dynamics in which institutional responses shape personal moral subjectivities. Indeed, the importance of recognition lies in its nature as a moral relationship – one that may be respected or violated [12,16].

From a more sociological perspective, military decorations thus appear to function as symbols embedded within complex systems of power and legitimacy. Drawing on Bourdieu’s theory of symbolic capital [17], decorations can be seen as tangible markers that confer legitimacy and even honor on recipients. Such symbolic capital, in turn, appears to influence social identity, shaping how people view themselves and how they are perceived in their community [18]. Even more so, as a form of recognition, it seems that decorations may reinforce a soldier’s sense of belonging and moral worth, offering validation that counters internal doubts or societal alienation [12,16]. However, research also shows that if expressions, however well-intentioned, are perceived as unjust, insufficient, or as instruments of institutional self-justification, they may exacerbate feelings of betrayal and alienation, deepening moral injury [12,16,19]. For instance, “many veterans actually feel alienated by pro-veteran societal praise” [2, p2] reporting that being hailed as heroes “made them feel extremely uncomfortable and aggravated their guilt” [10, p197].

### Relations between decorations and moral injury

Thus, decorations may both contribute to *less* and *more* moral injury. Regarding the attribution of blame to the system, again two conflicting expectations arise. On the one hand, if the soldier questions whether ‘the system’ acted justly, a decoration may exacerbate the situation as it can appear as if ‘the system’ is trying to cover up or justify unjust actions, which further proves that the system is wrong. This is evident in the opening quote, where the soldier deems the action wrong and expresses frustration that decorations were awarded for such a morally reprehensible event. On the other hand, bestowing a decoration upon the soldier may indicate to the soldier that, while the event itself was wrong, the actions of the specific soldier were indeed correct. This gesture might alleviate the soldier’s doubts regarding the justness of the system’s actions.

We set out to examine the effects of decorations on moral injury. The above-mentioned insights and associated expectations formed our hypotheses. For the specific preregistered hypotheses see the following link: https://osf.io/qk9rh/?view_only=600f248f5d8640ea848e7c03e324cbff. Our approach involved a combination of a quantitative experiment and qualitative interviews. First, we conducted a vignette-based experiment where participants encountered two scenarios (omission and commission) presented in a random order, accompanied by a moral injury scale [20] as well as specific questions concerning decorations. The scenarios were framed randomly toward self-blame or other/system-blame. Moreover, whether or not the participant received a decoration in the scenario was also randomized. None of our hypotheses were confirmed. However, the two scenarios did cause different levels of moral injury. Second, to gain preliminary qualitative insights into the effects of decorations and enhance interpretation, we conducted seven individual semi-structured interviews with (former) armed forces personnel.

## Materials and methods experiment

### Participants

For the experiment, we opted to broaden our participant pool beyond a specific subsection of the veteran population. We chose to recruit participants via online posts – LinkedIn – in favor of recruiting via VA or treatment centers, as the latter are rather specific subsections of the veteran population, which identify with the veteran status (which is not self-evident in the Netherlands) or suffer psychological problems. Moreover, we decided to invite civilians. Despite not being included in the main analyses, it may be useful for future research. For example, soldiers with combat experience tend to think that civilians (inside and outside of the armed forces) and soldiers without combat experience cannot imagine and differently experience the events they went through. Whether or not there is such a discrepancy is unknown. We sought to explore this. Moreover, the current study has an experimental and not a survey design, arguably for both armed forces personnel and civilians the vignettes would require them to imagine a situation they would not have been in before. Hence, we did not restrict who could fill in the survey. People could fill in the survey for approximately a month, from the 6th of July till the 4th of August 2023. We did not perform any power analysis as we could not with certainty determine how many participants we would get. Participants provided written consent. The data and materials are openly available in: https://researchbox.org/2374. The study received written approval from the Ethics Assessment Committee Faculty of Law and Nijmegen School of Management (reference 2019.04).

We obtained a gross sample of 516 participants, of which 150 participants did not work or study in the armed forces. We did not include the participants that did not work or study in the armed forces in the (main) analysis presented in this paper, but the data that is made available does include them for those interested. Of those employed by or studying in the armed forces (*N* = 366, 78.1% male), 46 (12.6%) served as citizens, one studied at the armed forces (0.3%), and 319 (87.2%) served as soldiers. Furthermore, 214 (76.7%) received decorations. For more details see Table 1. In conclusion, it is clear we have a wide and representative variety of participants.

**Table 1:** Demographics.

Answer option	Frequency	%	Answer option	Frequency	%
Branch			**Rank**		
Navy	35	9.6%	Flag officer	5	1.6%
Army	206	56.3%	Senior officer	73	23.8%
Airforce	31	8.5%	Subaltern officer	74	24.1%
Royal Marechaussee	37	10.1%	NCO	82	26.7%
Defense Support Command	35	9.6%	Corporal	34	11.1%
Command Material and IT	5	1.4%	Enlisted (excl NCO & corporal)	39	12.7%
General Staff	17	4.6%	Total	307	
Total	366				
**Deployment**			**Number of times deployed**		
No, I have never been deployed	86	23.6%	1 time	87	31.2%
Yes, I have been deployed	276	75.6%	2 times	77	27.6%
Yes, I am currently deployed	3	0.8%	3 times	39	14.0%
Total	366		4 times	32	11.5%
			More than 4 times	44	15.8%
			Total	279	

### Design and procedure

Participants, via an online questionnaire, read two scenarios, after each they were asked to fill in the 14-item Moral Injury Outcome Scale (MIOS) [20] and answer six questions formulated by us concerning decorations. Given the exploratory nature of the study, we asked participants to fill in their email addresses in case they were willing to participate in a *potential* follow-up interview. We took this step as a precaution in case the results would not be straightforward to interpret; which we made use of.

The first scenario was modeled on the Kabul evacuation. The second scenario concerned a firefight where civilian deaths occurred. The scenarios were presented in Dutch to participants but have been translated into English as best as possible for scrutiny, see Tables 2 and 3. The MIOS [20] was translated by the authors, aided by tools such as Google Translate. As the verb tense also had to be changed, back-translation did not make sense to us.

**Table 2:** Scenario 1: evacuation.

**Text** **vignette**	The following story is based on the experience of veterans. Imagine the following. You are part of an evacuation operation in an area torn by war. [Attribution of blame insert 1]. You were confronted with painful scenes on multiple occasions. A man with a desperate look in his eyes comes to you. He was holding a baby closely in his arms, begging for help. However, because of the strict security measures, they were not allowed to enter the airfield. In response, the man tried to hand over the baby to you. You took it over and tried to find a solution, but alas, it was for nothing and you had to give back the baby. It was your duty together with your colleagues to bring the evacuation to a good end and make sure that only those allowed to come along, went along. You saw the razor wire, the concertina, the border between you and safety. In an act of pure desperation, the man tried to throw the baby over the razor wire. The baby fell, via the crowd of people, on the ground. You don’t know what happened afterward, [Attribution of blame insert 2]. You have seen many women passed out and people who hurt themselves to get in. Both American and enemy combatants fired shorts to control the mass and people were beaten and mistreated. [Decoration condition insert]	
**Inserts**		
**Independent** **variable 1:** Attribution of blame	**Attribution of blame to self** **inserts:** Insert 1: [Empty] Insert 2: [because you had a short blackout from the stress]	**Attribution of blame to others/system inserts:** Insert 1: Even though the situation was severe in the area, you were sent to the area with too few resources and opportunities Insert 2: because you had to tend to another task
**Independent** **variable 2:** Decoration received	**No decoration condition:** [Empty]	**Received decoration condition:** You were awarded a decoration for your actions during this operation in the end. You helped many people but also weren’t able to help many others

**Table 3:** Scenario 2: firefight.

**Text** **vignette**	The following story is based on the actual experience of veterans. Imagine the following. You are deployed to the Middle East for a safety mission. During this counterinsurgency mission you are oftentimes confronted with guerilla fighters (insurgents). At one point, your unit is called upon to find enemy combatants who are hiding in a small village. This resulted in a firefight. The enemy combatants were shooting at you with heavy weapons from a residency. Because of the suppression, you were not immediately able to move forward toward the other enemy combatants who were spread around nearby buildings. You regrouped behind a small wall and, whilst awaiting air support, called in mortar support. This hit the target within a few minutes. After a couple of direct hits (FFE) the suppression disappeared as well as any resistance shortly after. [Attribution of blame insert] Afterwards it appeared that the building from which the heavy weapons fired also had civilians in it. A mother and a daughter (4) died from the mortar fire. [Decoration condition insert]	
**Inserts**		
**Independent** **variable 1:** Attribution of blame	**Attribution of blame to self insert:** There probably were no civilians in the residency but you can never be certain. Nevertheless, you called in (mortar)fire on the combatants in the residency	**Attribution of blame to others/system insert:** You heard on the radio from the leaders at the camp (the OPS) that there were no civilians in the residency. Hence, you called in (mortar) fire on the combatants in the residency.
**Independent** **variable 2:** Decoration received	**No decoration condition:** [Empty]	**Received decoration condition:** You were awarded a decoration for your actions during this operation in the end. You eliminated enemy soldiers but there were also civilian casualties.

The MIOS [20] was scored on a 5-point scale (0 = Strongly disagree; 4 = Strongly agree). Additionally, we constructed four variables related to decorations ourselves (Earned, Pride, Confrontation, and Memories). These four variables were scored on the same scale as the MIOS. They respectively concerned whether the participant felt (s)he earned the decoration, is proud about having been awarded the decoration, finds the decoration confronting, and if (s)he thought it would bring up memories of the victims. Furthermore, we constructed two variables respectively concerning where participants would store the decoration and to whom they would talk to about the events. We are not aware of any validated scales for these purposes, hence, we had to make our own exploratory questions to get some first indications.

Participants were assigned to one of four conditions per scenario. The four conditions were based on two variables. Following the afore-discussed distinction between self-blame and blaming others, the first variable was designed to make participants either blame him/herself (*attribution of blame to self*) or someone else (*attribution of blame to others/system*). The second variable concerns whether participants received a decoration in the scenario or not. This yields four conditions per scenario, SEN = self-attribution no decoration received, SER = self-attribution decoration received, SYN = system attribution no decoration received, and SYR = system attribution decoration received. Note that scenarios 1 and 2 were respectively designed around the distinction between omission (i.e., moral injury due to one’s inactions) and commission [5] (i.e., moral injury due to one’s actions).

## Results and discussion experiment

### Confirmatory factor analysis

To determine an acceptable fit of the MIOS [20], we used the Chi-square (*p* ≥ .05 is acceptable), RMSEA (≤.05 indicates a good fit,.05 ≤ RMSEA < .10 indicates an acceptable fit, RMSEA ≥ .10 indicates an insufficient fit), CFI (≥.90 indicates an acceptable fit) and TLI (≥.90 indicates an acceptable fit). In both scenarios, the CFA confirmed the two-factor structure. For the first scenario: RMSEA = 0.080, CFI = 0.918 and TLI = 0.901. The *p*-value of the Chi-square, however, is significant, χ2 (76, **N* *= 516) = 329.527, *p* < .001. Nonetheless, the results together confirm the two-factor structure. For the second scenario: RMSEA = 0.080, CFI = 0.936 and TLI = 0.923. The *p*-value for the Chi-square, however, again is significant, χ2 (76, *Ν* = 484) = 314.335, *p* < .001.

### Confirmatory analyses

We deviated from the preregistered exclusion criteria by excluding participants who did not answer all questions of the MIOS [20] for the analyses concerning H1-H4, as we needed participants to answer all of these questions to get a correct sum score (original criteria were only that participants had to answer whether they worked or studied for the Dutch armed forces). For scenario 1, five additional participants were excluded; for scenario 2 no additional participant was excluded for this reason. Given the total number of participants, this deviation is unlikely to have a major impact on the results. For the other analyses, not all participants answered all questions, hence there is some fluctuation in the degrees of freedom of the analyses.

As can be seen in Table 4, we found no differences between the experimental conditions within the scenarios in terms of moral injury (alpha = .05). Hypotheses 1–4 therefore received no support. With respect to our constructed decoration variables, one test for hypothesis 6 was significant (*earned*) in scenario 1 but not in scenario 2, which leads us to conclude that hypothesis 6 was not supported by the data. Hypothesis 6 was a comparison in the self-attributed blame condition between those who did and those who did not receive a decoration. None of the other tests for hypotheses 5–8 were significant, see S1 Table.

**Table 4:** Confirmatory Hypotheses Moral Injury.

Scenario 1: evacuation												
		SEN		SER		SYN		SYR		*t*	*df*	*p*
		*Min =* 2 *Max =* 43		*Min =* 5 *Max =* 42		*Min* = 0 *Max* = 46		*Min* = 5 *Max* = 44				
	Outcome	*M*	*SD*	*M*	*SD*	M	SD	M	SD			
H1	Moral Injury	21.23	8.31			22.74	8.13			-1.268	189	.206
H2	Moral Injury	21.23	8.31	22.39	8.25					-0.957	185	.340
H3	Moral Injury					22.74	8.13	22.63	8.29	0.092	177	.927
H4	Moral Injury			22.39	8.25			22.63	8.29	-0.188	173	.851
**Scenario 2: firefight**												
		SEN		SER		SYN		SYR		*t*	*df*	*p*
		*Min =* 0 *Max =* 40		*Min =* 0 *Max =* 34		*Min =* 2 *Max =* 32		*Min =* 0 *Max =* 36				
	Outcome	*M*	*SD*	*M*	*SD*	*M*	*SD*	*M*	*SD*			
H1	Moral Injury	17.99	8.17			17.49	7.13			0.434	175	.665
H2	Moral Injury	17.99	8.17	16.48	8.36					1.190	167	.236
H3	Moral Injury					17.49	7.13	16.56	8.32	0.793	172	.429
H4	Moral Injury			16.48	8.36			16.56	8.32	-0.065	164	.948

*Note.* SEN = self-attribution, no decoration received condition; SER = self-attribution, decoration received condition; SYN = system-attribution, no decoration received condition; SYR = system-attribution, decoration received condition.

Item-level information on the MIOS [20] can be found in S2 Table, which includes the item wording (both the original and the translated version). In both scenarios, item 6 (i.e., disgust by what happened; *M*_scenario 1_ = 3.08, *M*_scenario 2_ = 2.34) and item 9 (i.e., losing the belief that there is a higher power; *M*_scenario 1_ = 2.16, *M*_scenario 2_ = 1.94) scored particularly high (range = 0–4). It thus appears that participants condemned the presented situations (item 6). Moreover, item 9 may be particularly high because of the irreligiousness of the Dutch population. Indeed, some participants complained in the comments section at the end of the survey about the religious nature of the 9th item.

Furthermore, the Chi-square test indicated that there is no association between the experimental condition and where the decoration is stored for either scenario, for scenario 1: χ2(12) = 5.996, *p* = .916, for scenario 2: χ2(12) = 11.332, *p* = .502. For the frequencies and percentages, see S3 Table. Against what was preregistered, we did not conduct a multinomial logistic regression for the event talking variable as it concerns multi-response instead of nominal data. Instead, we have reported the frequencies and percentages in S4 Table. The results indicate that there are little to no differences between the conditions in who would be talked to about the events.

### (Preregistered) exploratory analyses

As per the preregistration, we also conducted multiple exploratory analyses. Firstly, we compared the four conditions on the factor scores instead of the total moral injury score. In none of these comparisons was there a significant difference. Furthermore, we performed an exploratory factor analysis on the four metric decoration variables (*Earned, Pride, Confrontation, and Memories*). The results favor a one-solution approach (for the Scree Plots, see S1 and S2 Figs). For scenario 1, the eigenvalues are (using a principal component analysis): 2.666, 0.720, 0.340, and 0.275, for scenario 2 the eigenvalues are 2.603, 0.808, 0.305, and 0.286 respectively for components 1–4. This scale appears to follow the moral injury scores quite strongly (scenario1: *r*(356) =.466, *p* < .001, scenario 2: *r*(339) =.593, *p* < .001, note that these correlational analyses were not preregistered). Lastly, participants who did not work at the armed forces (scenario 1: *M* = 26.9, *SD* = 9.9, scenario 2: *M* = 22.6, *SD* = 10.4) had higher moral injury scores than participants who worked as citizens for the armed forces (scenario1: *M* = 24.5, *SD* = 7.4, *t*(194) = 1.737, *p* = .086, scenario 2: *M* = 20.1, *SD* = 6.6, *t*(181) = 1.833, *p* = .070), who in *t*urn scored higher *t*han soldiers at the armed forces (scenario 1: *M* = 21.9, *SD* = 8.3, *t*(363) = 2.069, *p* = .039, scenario 2: *M* = 16.7, *SD* = 8.1, *t*(340) = 2.618, *p* = .003).

An analysis we did not preregister is a comparison between the scenarios. Nonetheless, we did perform it as an exploratory analysis. There is a significant difference between the moral injury score on the first and second scenarios, whereby the first scenario leads to more moral injury than the second scenario (*M*_scenario 1_ = 22.11, *M*_scenario 2_ = 17.15, *t*(342) = 12.495, *p* < .001). The effect is medium to large (*d* = 0.68).

## Discussion

The results of the experiment indicate that moral injury is not influenced by a) whether one receives a decoration or not, b) whether one blames oneself or others. However, what does seem to matter is whether the moral injury-inducing event is due to action or inaction, whereby inaction (i.e., scenario 1) leads to more moral injury than action (i.e., scenario 2). The distinction between commission and omission (i.e., action vs. inaction) made by Litz et al. [5] therefore seems quite relevant. One of the main questions, of course, is whether the null findings concerning our main hypotheses reflect reality or if they are purely a product of methodological choices. One of our goals was to see whether a quantitative experimental approach is able to capture differences in moral injury. A major argument in favor of this concerns causality, as only an experimental approach is able to prove causality.

Criticism could be levelled against our approach to use a vignette-based experimental design. One such critique concerns the hypothetical nature of vignette-based experiments. Indeed, it seems reasonable to argue that the majority of participants, even those with combat experience, are unlikely to have experienced either scenario before [21] which could make it difficult for them to accurately indicate how they would feel. However, it is important to note that hypothetical scenarios do not necessarily lead to different conclusions than actual experiences (cf. [22] in the case of forgiveness). Moreover, previous research has shown that hypothetical stories in vignettes can lead to the expression of moral emotions that are part of moral injury, such as anger [23,24]. Furthermore, during the review process, we eventually came across a single other study on moral injury that used an experimental vignette design (in a military context) [25]. As they note “experimental subjects in a lab setting are not morally injured. Thus, one can only create mildly stressful MI conditions to conduct controlled studies” [25, p9] and continue “However, we suggest that these differences are a matter of degree and not of kind” [25, p9].

The quote at the end of the previous paragraph also indicates that the means reported in our study are appropriate for the design. Indeed, our means are somewhat lower but still comparable to those reported in the study validating the MIOS scale [20] (see Table 3 in their study). Moreover, the means reported by the previously mentioned other experimental study, are also below the midpoints of their scales [25]. Lastly, high means on moral injury indicators would not be preferable for an experimental study to begin with. In a survey, researchers merely measure moral injury already present within participants, in contrast, experimentally inducing high levels of moral injury would be highly ethically questionable.

Nonetheless, one way to potentially make it more likely that participants can accurately predict how they would feel is to immerse them more into the situation. This could be achieved by using audio and/or visual material [26]. One study used virtual reality to present a scenario to nurses [27]. Another used audio to immerse students into experiencing a traffic accident [28]. Both experimental manipulations seemed to have worked reasonably well but there is currently no evidence that these methods actually elicit more genuine responses. Moreover, making audio or video-based vignettes requires substantially more resources than standard text-based vignettes. Hence, more research is needed to make conclusions about the validity of the different ways to present experimental material. In conclusion, the approach used here does appear to be valid for the time being, even if improvements can be made. To shed more light on the findings in our experiment, we followed up with interviews.

### Materials and methods interviews

At the end of the experiment, we invited participants to indicate their willingness for a follow-up interview; five agreed, and several others contacted us independently via email. In total, we interviewed 7 interviewees. Using maximum variation sampling [29], we selected participants based on gender (2 female, 5 male), MIOS score (2 below the midpoint, 3 above, 2 non-participants), deployment status (6 deployed, 1 not), rank (3 senior officers, 3 NCOs, 1 enlisted), and receipt of individual commendation (3 had received a medal for personally commendable actions). Deployment contexts included Iraq (1990–1991), the former Yugoslavia (1992–2002), Afghanistan (2015–2021), and Somalia (2008–present), with a slight focus on the Yugoslav context. Some had been deployed once, others several times. Although we did not select participants based on their branch, there was a wide variety. While some participants initially stated they had not been decorated, all had received at least a deployment medal, resulting in no interviewees without any form of recognition. Thus, the nature and significance of the decorations received by the interviewees differed considerably.

Interviews were semi-structured, which enabled us to hone in on our research questions and hypotheses while at the same time allowing the interviewees to bring up what they felt was important [30]. The topics covered moral injury, preparation and aftermath of deployment, the role of decorations, and contextual factors. Participants also reflected on the experimental results. Interviews ranged from 45 minutes to two hours, shaped by the depth of participants’ experience.

Thematic analysis was conducted, which involves an emergent process of interpretation with some thematic structure as the typical outcome [30,31]. Two researchers reviewed notes and transcripts, generated preliminary codes, and refined these through discussion. This iterative process helped ensure analytic coherence. Emerging themes were analyzed in relation to our theoretical framework and experimental findings. In line with this, we adopted a selective transcription strategy: segments relevant to our analysis were transcribed verbatim, allowing for accuracy where it mattered most [32]. Table 5 summarizes the results of the thematic analysis.

**Table 5:** Themes qualitative interviews.

Fragments of the interviews	Focused codes	Overarching codes
1. “more as a kind of certificate, that you were there, that you participated in it [the operation], and not so much as indicating pride that it has been something successful”. 2. [concerning the news of Srebrenica] “Everyone in the Netherlands then criticized Dutchbat … When all the facts are brought to light, only then do you get recognition 25 years later. Even though there are people who have justly been struggling with psychological issues for 25 years because of it. Then a medal, in first instance, definitely does not add anything, in fact, it only makes it worse.”	Decorations as recognition (erkenning) versus appreciation (waardering)	The perceived moral significance of decorations
1. “These are not the standard stories people just throw on the table ... and sometimes they also lead to a conversation with the colleague, asking where have you been and which missions did you do?”	Decorations as in-/exclusion among colleagues	
1. [Concerning values that decorations carry] “Also that you can pass something on to the next generations and then I mean, for example, I now have grandchildren, I think it is good for younger generations who are grown up in a very stable situation, to realize that it is not self-evident. 2. “I find it [decorations] to be a very important signal to civil society not to forget that … war is of all times. And the only thing we can do is for there to be people in society who are prepared to get themselves into harm’s way to protect what we have built up together.”	Decorations as communication to society	
1. “It is not that the military wants to say with it [decorations] that everything you did there was immediately good … It is specifically the approval of the colleagues among each other” 2. “I did what I did there and I know what I did there. I don’t need a medal to recognize what I did. My story, it is in itself my badge of honor, and when I talk to people about what I did there, that’s my badge of honor.	Role of peers versus leaders	The role of context in the perceived moral significance of decorations
1. “Yes, they wanted to give us that medal to recognize that we did something that was not normally done, but me not being able to talk about it, or show it, or display it, or anything like that, kind of gives me the impression that they did it just to say: ‘[name participant], good job! Thanks, but don’t say anything. So it was like a medal for appeasement. In recognizing that, the medal means nothing to me now.”	Role of organization	
1. [Concerning how Srebrenica was portrayed on the news] “I watched TV nail-biting and with a red head in the summer of 95. …I think that it [misrecognition by society and the media] is a bigger factor in PTSD than the actual being under fire and doing combat actions.” 2. “I think many civilians in civil society don’t know what they are talking about. They are not in such a situation, they don’t even know what such a decoration means when they see it … they don’t know what the person has been through” 3. “I think every soldier should be valued for what we do because it isn’t normal what we do … and people may not even have that realization. So on the one hand I think we deserve that recognition. Only it does make you recognizable, which allows people to make a judgment about you without knowing what it does with you as a person.”	Role of societal perceptions	

## Results and discussion interviews

### The perceived moral significance of decorations

The qualitative semi-structured interviews we conducted helped to interpret and further enrich our understanding of the experiment’s results. One prominent theme that emerged from the interviews is the nuanced nature of the significance associated with decorations. While there is a general consensus on the moral values required to merit a decoration (e.g., courage, selflessness), the moral message conveyed by the decorations themselves turned out to be less straightforward for participants.

### Decorations as recognition versus appreciation

The interviews revealed a consensus that decorations primarily signify recognition (Dutch: erkenning), although there were variations in their interpretation of this meaning. All participants agreed that decorations serve as a form of acknowledgment, such as the factual recognition of an individual’s involvement in a specific military operation and acknowledgment of their efforts in challenging circumstances. However, not all participants believed that decorations necessarily imply appreciation (Dutch: waardering) in the sense of justification or approval of one’s actions. For example, one participant distinguished between decorations awarded simply for mission participation without any negative behavior and those given for specific actions. This participant argued that only the latter type of decorations conveys both acknowledgment and appreciation, stating that he viewed it “more as a kind of certificate, that you were there, that you participated in it [the operation], and not so much as indicating pride that it has been something successful”.

### Decorations as in-/exclusion among colleagues

In all cases, decorations clearly have moral meaning, thereby carrying social significance as well, serving social-signaling functions. As participants observed, decorations distinguish individuals who participated in specific deployments or had particular experiences, such as combat. Consequently, decorations serve as both inclusive and, inevitably, exclusive markers. For example, in the Netherlands, there was recent discussion about whether soldiers sent to Lithuania should qualify for a mission decoration, with soldiers feeling overlooked due to the operation not being classified as an official military deployment. Simultaneously, it is due to these exclusionary effects that decorations distinguish soldiers who do receive such recognition. Additionally, as noted by one participant, decorations can serve as conversation starters among soldiers. Even if soldiers might be hesitant to share their experiences with new colleagues, wearing their medals during special occasions accomplishes that for them: “These are not the standard stories people just throw on the table... and sometimes they also lead to a conversation with the colleague, asking where have you been and which missions did you do?” Thus, decorations possess the dual social potential to both set soldiers apart and foster cohesion among them.

### Decorations as communication to society

Participants furthermore emphasized the societal significance of decorations, highlighting their role as tangible memories of events, not only for the soldiers themselves but also for society at large. One participant mentioned that decorations serve as a means of communicating to society the reality of war and the dedicated efforts of soldiers to assist those affected by it. Consequently, decorations were perceived not only as memorials but also as educational tools and external justifications toward society.

Notably, the varying interpretations were, in part, influenced by the specific type of decorations participants had in mind. This includes the distinction between a generic decoration, received by every soldier after completing a military deployment of more than 30 days, and a distinctive and rare medal awarded for displaying exceptional courage in a specific action. Moreover, the interpretations also appeared to depend on factors such as the circumstances surrounding the decoration’s presentation and the soldier’s experience of their actions and the associated decoration, as well as other contextual factors. The following section discusses the role of context in the perceived moral significance of decorations.

## The role of context in the perceived moral significance of decorations

### Role of peers versus leaders

In the military, solitary actions are a rarity. Accordingly, when asked to reflect on our hypothesis about how decorations might mitigate moral injury, some participants invoked the role of their colleagues, emphasizing the significance of their judgment compared to that of high-ranking officials who distribute decorations. One participant for instance asserted that their moral assessment of an event “specifically has to do with your own feelings and those of your colleagues”, while a decoration “has nothing to do with whether you acted good or bad”. As stated, while all participants agreed that decorations convey acknowledgment of what you did, some found justification and approval are distinct. In the words of the same participants: “It is not that the military wants to say with it [decorations] that everything you did there was immediately good”. Instead, they emphasized that “it is specifically the approval of the colleagues among each other” that holds significance. Participants stressed that the endorsement from their comrades surpasses the importance of decorations awarded by the military organization (after a significant passage of time and requiring self-application). Combat veterans were particularly adamant about this, asserting that the colleagues who were present during the actions, are better positioned to assess them compared to higher-ranking officials. So, the interpretation of a soldier’s decoration is significantly influenced by the specific context surrounding the events for which they are honored, as well as the identity of the military actors expressing their appreciation to the soldier.

### Role of organization

The participants’ accounts also point to the critical aspect of how the decoration is awarded by the military organization. For instance, one participant shared a notably negative experience associated with the entire process of receiving a specific decoration. In this instance, the decoration was conferred in private and in an impersonal manner, resulting in the antithesis of the intended effect – rather than feeling appreciated, the soldier experienced a distinct lack of acknowledgment. This experience highlights the need to attend to the specific context of the decoration process, and the organizational context more generally.

### Role of societal perceptions

The wider societal context appeared as a final important theme. When queried whether feeling valued and recognized by a decoration becomes more difficult when a specific mission is portrayed negatively in the media and society, responses varied. Some participants indicated that such negative portrayals could indeed hinder their sense of value and recognition, particularly for decorations awarded based on the duration of a mission. Conversely, others argued that societal judgment of them should not hinge on the justice or injustice of the mission itself but rather on individual behavior during the mission. That said, in both cases participants expressed dissatisfaction or even frustration with how the media covers military actions.

### Can decorations positively or negatively influence moral injury?

The interviews indicate that decorations may have a discernible effect, albeit small. Positively, while the opinions of fellow service members are deemed more crucial, decorations are generally experienced as conveying recognition or even appreciation. In this regard, they may play a constructive role in the long term for soldiers reflecting, grappling with doubts, and coping with events. Conversely, on the negative side, if decorations are perceived as insincere or even morally inappropriate, they may have adverse consequences, including the aggravation of soldiers’ moral injuries.

Implicit in our reasoning in the introduction for why decorations may have an effect on moral injury, positively or negatively, is that we assumed decorations to inherently carry a sense of moral approval. The interviews, however, indicate that this belief is not ubiquitously shared among participants. Some participants did indeed see (some) decorations as signaling appreciation or approval whereas others saw them merely as factual acknowledgement. This could be a potential individual-level moderator influencing whether or not decorations affect moral injury. Moreover, this distinction seems to (partly) intertwine with the distinction between decorations awarded for mission participation compared to those awarded for specific actions, with the latter being more likely to carry moral significance. It is in any case something future research should make explicit to participants when presenting a scenario as this could be an important contextual moderator.

The interviews thereby help us in reinterpreting the null findings observed in the experiment. Together the quantitative and qualitative results indicate that decorations probably have a small effect when they are considered to carry moral approval by an individual. However, more research is needed to draw any firm conclusions.

## Conclusion

Although decorations played a relevant role in the war stories Shay [1] heard from soldiers, there has been no research that we are aware of that investigated a possible relation between decorations and moral injury. As noted by Molendijk et al. [2], the social phenomena of recognition and rituals, such as being awarded a decoration, have received little attention in moral injury research. To our knowledge, no research has yet investigated the actual effects of rituals on moral injury.

In this article, we report an experiment that investigated the effects of receiving a decoration on moral injury through two scenarios and supplemented by semi-structured interviews. The first scenario concerned an evacuation; the second scenario involved a firefight. We manipulated whether the participant in each scenario received a decoration or not. Furthermore, we manipulated the framing of who was to blame, the person him/herself or the system/trusted others. Our quantitative results indicate that receiving a decoration does not significantly impact moral injury. None of the experimental conditions were (systematically) different on the dependent variables (moral injury scale and six variables related specifically to decorations). An exploratory analysis did however reveal a significant difference between the first (in which participants failed to prevent civilian deaths) and second (in which participants caused civilian deaths) scenarios. Conversely, the interviews indicated that decorations may have either a positive or negative effect, depending on the situation. This result is a first step toward experimentally differentiating events in terms of the severity of moral injury they are expected to cause and the role of decorations, a possible social-cultural way of healing.

Research on moral injury indicates that recognition plays an important role in shaping individuals’ experiences [10,11]. But what is recognition? The exploration of this concept delves into a complex social phenomenon, having prompted entire fields in philosophy and the social sciences to dedicate themselves to the topic (e.g., [12,13,33]). The question of what constitutes recognition is multifaceted. Is recognition, as a participant indicated, a mere factual statement of what happened, or is it something else? And what does it mean to participants? As Molendijk notes “recognition does justice to one’s experiences, while misrecognition does injustice to one’s experiences and as such can be morally injurious” [11 p319]. In any case, research on these themes suggests that recognition should not be equated with praise but rather with validation. Recognition is the act of validating one’s own interpretation of one’s experience, providing a sense of understanding [12,13,33]. As a result, for individuals struggling with events they perceive as unjust, receiving an award as a sign of praise may paradoxically be experienced as misrecognition rather than recognition. This complexity may explain why decorations may not have a direct effect on moral injury in many cases. It may further lead us to hypothesize that decorations can be perceived as a helpful sign of recognition for those unsure of the legitimacy of their actions. Conversely, they may cause a feeling of misrecognition for individuals who unequivocally experience guilt and/or shame, thereby exacerbating feelings of betrayal and shame. The net effect, as observed in our study, may average out to zero.

To state it explicitly, we argue that recognition and appreciation mediate the relationship between decorations and moral injury. Decorations as such are mere pieces of metal and cloth. Only in their function of signaling something, such as recognition, appreciation, or veteran status, do they have meaning. As recognition and appreciation play an important role in moral injury [10,11], it follows that decorations can alleviate or exacerbate moral injury. This insight may be useful for policymakers in designing their policies on decorations. Similarly, it may prove a useful talking point for clinicians.

Furthermore, it seems pertinent to make a distinction between decorations, specifically between those awarded for fulfilling a deployment duration without misconduct and those bestowed for demonstrating specific behaviors like bravery or courage. These can be respectively referred to as omission decorations and commission decorations. The former signifies the general avoidance of disapprovable behavior, conveying a form of acknowledgment without necessarily implying approval. In contrast, the latter is exclusively granted when behavior surpasses a higher-than-ordinary standard, automatically signifying the explicit approval of the actor granting the decoration. The difference between the two then at least concerns two dimensions: a lack of disapproval versus explicit approval, and one’s overall behavior during deployment versus a specific situation during deployment. Thus, the moral judgment conveyed through both decorations, and thus the type of recognition they represent, is different.

What we also saw in the experiment and an interview was that not acting felt worse than acting. As this was rather shortly discussed and not covered in the other interviews, we did not elaborate on it in the results section. While an academic may say that doing nothing is also a decision, that is not how our participant saw it. One joins the military to take action, not to stand back and watch. This may explain why the variable Earned was almost significantly higher in the SER condition than the SYR condition in scenario 1 but not in scenario 2, and why scenario 1 resulted in higher moral injury than scenario 2. A decoration may not be perceived as needed but if the military organization decides to award it, it is appreciated. Future research should investigate this further.

A noteworthy element is that soldiers have to nominate themselves for commission decorations (as we have termed them). Hence, it may be reasonable to assume that participants saw the decoration received conditions as situations in which they applied for the decoration themselves and thus obviously felt they earned it, whereas they did not apply for it in the decoration not-received conditions. However, we do not see a systematic difference between these two types of conditions over the scenarios and in all such comparisons. Hence, to what extent this is actually the case, remains unclear.

Lastly, being a socio-cultural artefact, what holds in one national-military context, may not hold in another military context. For example, Gavriely-Nuri’s [14] research was conducted in Israel, whereas our research was conducted in the Netherlands. Despite there being similarities, differences may also manifest themselves. For example, the Netherlands being a less religious country than Israel, the spiritual aspect of moral injury and decorations may be different between the two countries. Further research could look into controlling for these and other socio-cultural factors, such as the role of peers, leaders, and society at large, which appeared to be relevant factors in the interviews.

Thus, assuming the results at least partially reflect reality, they offer support for and refinement of the theoretical framework, by demonstrating how moral injury is not only shaped by internal psychological mechanisms but is embedded within broader moral relationships and institutional dynamics [11,12,17]. In line with Shay’s original emphasis on the social roots of moral injury [1], the interviews reveal how, while current clinical models often foreground the individual’s internal experience [7–9], participants repeatedly pointed to the role of institutional recognition (or its absence) in shaping moral self-assessments, aligning with research on institutional betrayal and recognition [15–16]. On the one hand, the data suggests that decorations, when offered in a manner perceived as authentic and congruent with peer recognition, may offer a form of symbolic capital [17], restoring a sense of honor and belonging [18]. On the other hand, decorations that were perceived as insincere, routine, or detached from lived experience risked reinforcing feelings of alienation and injustice, echoing the notion that symbolic acts can exacerbate rather than repair moral injury if misaligned with the recipient’s moral understanding [12,19]. Thus, when decorations are understood as mere bureaucratic gestures, devoid of deeper moral engagement, they appear to fail to confer symbolic capital and do not contribute to a positive social identity.

In essence, the nuanced nature of these distinctions underscores the complexity of the effects of decorations on moral injury. Through this study, we took the first step toward a comprehensive investigation of these effects.

## Supporting information

S1 Table Confirmatory Hypotheses Decoration Variables. (DOCX)

S2 Table Item-level Descriptive Statistics (MIOS). (DOCX)

S3 Table Decoration Display Frequencies and Percentages. (DOCX)

S4 Table Event Talking Frequencies and Percentages. (DOCX)

S1 Fig Scree Plot Exploratory Factor Analysis Scenario 1. (DOCX)

S2 Fig Scree Plot Exploratory Factor Analysis Scenario 2. (DOCX)
