# Supplementary material for: Types of decorations, their social meaning and influence on moral injury: A mixed methods approach
Source: PLoS One. 2025 Oct 27;20(10):e0333344. doi: 10.1371/journal.pone.0333344 (PMC12558466; doi:10.1371/journal.pone.0333344)
Supplement: S1 Table — (DOCX) [file pone.0333344.s001.docx]

**S1 Table.** **Confirmatory Hypotheses Decoration Variables.**

| Scenario 1 | | | | | | | | | | | | |
| --- | --- | --- | --- | --- | --- | --- | --- | --- | --- | --- | --- | --- |
|  |  | SEN | | SER | | SYN | | SYR | | *t* | *df* | *p* |
|  |  | *M* | *SD* | *M* | *SD* | *M* | *SD* | *M* | *SD* |  |  |  |
| H5 | Earned | 1.98 | 1.01 |  |  | 1.96 | 1.06 |  |  | 0.143 | 187 | .886 |
|  | Pride | 2.28 | 1.08 |  |  | 2.03 | 1.13 |  |  | 1.570 | 187 | .118 |
|  | Confront. | 1.96 | 1.22 |  |  | 2.03 | 1.17 |  |  | -0.428 | 186 | .669 |
|  | Memories | 2.05 | 1.07 |  |  | 2.03 | 1.07 |  |  | 0.133 | 187 | .894 |
| H6 | Earned | 1.98 | 1.01 | 2.30 | 0.92 |  |  |  |  | -2.270 | 182 | .024 |
|  | Pride | 2.28 | 1.08 | 2.13 | 1.13 |  |  |  |  | 0.917 | 182 | .360 |
|  | Confront. | 1.96 | 1.22 | 2.17 | 1.07 |  |  |  |  | -1.244 | 181 | .215 |
|  | Memories | 2.05 | 1.07 | 2.16 | 1.07 |  |  |  |  | -0.666 | 182 | .506 |
| H7 | Earned |  |  |  |  | 1.96 | 1.06 | 2.02 | 1.01 | -0.428 | 173 | .669 |
|  | Pride |  |  |  |  | 2.03 | 1.13 | 2.10 | 1.13 | -0.391 | 173 | .696 |
|  | Confront. |  |  |  |  | 2.03 | 1.17 | 2.20 | 1.05 | -0.978 | 173 | .329 |
|  | Memories |  |  |  |  | 2.03 | 1.07 | 2.33 | 1.04 | -1.883 | 173 | .061 |
| H8 | Earned |  |  | 2.30 | 0.92 |  |  | 2.02 | 1.01 | 1.879 | 168 | .062 |
|  | Pride |  |  | 2.13 | 1.13 |  |  | 2.10 | 1.13 | 0.208 | 168 | .835 |
|  | Confront. |  |  | 2.17 | 1.07 |  |  | 2.20 | 1.05 | -0.178 | 168 | .859 |
|  | Memories |  |  | 2.16 | 1.07 |  |  | 2.33 | 1.04 | -1.090 | 168 | .277 |
|  |  |  |  |  |  |  |  |  |  |  |  |  |
| Scenario 2 | | | | | | | | | | | | |
|  |  | SEN | | SER | | SYN | | SYR | | *t* | *df* | *p* |
|  |  | *M* | *SD* | *M* | *SD* | *M* | *SD* | *M* | *SD* |  |  |  |
| H5 | Earned | 2.28 | 0.90 |  |  | 2.16 | 0.98 |  |  | 0.867 | 174 | .387 |
|  | Pride | 2.33 | 0.89 |  |  | 2.23 | 1.06 |  |  | 0.624 | 174 | .533 |
|  | Confront. | 1.92 | 0.99 |  |  | 1.84 | 1.14 |  |  | 0.461 | 174 | .646 |
|  | Memories | 2.17 | 1.02 |  |  | 2.10 | 1.04 |  |  | 0.479 | 174 | .632 |
| H6 | Earned | 2.28 | 0.90 | 2.26 | 1.03 |  |  |  |  | 0.154 | 166 | .878 |
|  | Pride | 2.33 | 0.89 | 2.29 | 0.99 |  |  |  |  | 0.227 | 166 | .820 |
|  | Confront. | 1.92 | 0.99 | 1.87 | 1.10 |  |  |  |  | 0.328 | 166 | .743 |
|  | Memories | 2.17 | 1.02 | 2.04 | 1.14 |  |  |  |  | 0.828 | 166 | .409 |
| H7 | Earned |  |  |  |  | 2.16 | 0.98 | 2.43 | 0.94 | -1.900 | 171 | .059 |
|  | Pride |  |  |  |  | 2.23 | 1.06 | 2.34 | 0.94 | -0.680 | 171 | .497 |
|  | Confront. |  |  |  |  | 1.84 | 1.14 | 1.80 | 1.03 | 0.297 | 171 | .767 |
|  | Memories |  |  |  |  | 2.10 | 1.04 | 2.00 | 1.07 | 0.623 | 171 | .534 |
| H8 | Earned |  |  | 2.26 | 1.03 |  |  | 2.43 | 0.94 | -1.159 | 163 | .248 |
|  | Pride |  |  | 2.29 | 0.99 |  |  | 2.34 | 0.94 | -0.298 | 163 | .766 |
|  | Confront. |  |  | 1.87 | 1.10 |  |  | 1.80 | 1.03 | 0.426 | 163 | .671 |
|  | Memories |  |  | 2.04 | 1.14 |  |  | 2.00 | 1.07 | 0.213 | 163 | .832 |

*Note.* SEN = self-attribution, no decoration received condition; SER = self-attribution, decoration received condition; SYN = system-attribution, no decoration received condition; SYR = system-attribution, decoration received condition.
