# Supplementary material for: Types of decorations, their social meaning and influence on moral injury: A mixed methods approach
Source: PLoS One. 2025 Oct 27;20(10):e0333344. doi: 10.1371/journal.pone.0333344 (PMC12558466; doi:10.1371/journal.pone.0333344)
Supplement: S2 Table — (DOCX) [file pone.0333344.s002.docx]

**S2 Table. Item-level Descriptive Statistics (MIOS).**

|  | Scenario 1 | | Scenario 2 | |  |
| --- | --- | --- | --- | --- | --- |
| Item | **Mean** | **SD** | **Mean** | **SD** | **Item wording (Dutch is used in experiment, English is the original)** |
| 1 | 1.72 | 1.006 | 1.27 | 1.006 | **NL:** Ik zou mezelf de schuld geven  **ENG:** I blame myself. |
| 2 | 1.98 | 0.995 | 1.15 | 0.815 | **NL:** Ik zou mijn vertrouwen in de mensheid verliezen  **ENG:** I have lost faith in humanity. |
| 3 | 1.26 | 0.918 | 1.13 | 0.882 | **NL:** Ik zou gaan denken dat mensen me zouden haten als ze me écht zouden kennen  **ENG:** People would hate me if they really knew me. |
| 4 | 1.76 | 1.019 | 1.30 | 0.910 | **NL:** Ik zou het moeilijk vinden om het goede in anderen te zien  **ENG:** I have trouble seeing goodness in others. |
| 5 | 1.18 | 0.802 | 1.03 | 0.723 | **NL:** Ik zou gaan denken dat mensen geen tweede kans verdienen  **ENG:** People don’t deserve second chances. |
| 6 | 3.08 | 0.882 | 2.34 | 1.096 | **NL:** Ik zou walgen van wat er gebeurd was  **ENG:** I am disgusted by what happened. |
| 7 | 1.09 | 0.886 | 1.05 | 0.841 | **NL:** Ik zou het gevoel hebben dat ik geen goed leven verdien  **ENG:** I feel like I don’t deserve a good life. |
| 8 | 1.26 | 0.941 | 1.06 | 0.847 | **NL:** Ik zou mezelf gaan belemmeren in het hebben van succes  **ENG:** I keep myself from having success. |
| 9 | 2.16 | 1.238 | 1.94 | 1.212 | **NL:** Ik zou gaan denken dat er geen hogere macht is  **ENG:** There is no higher power. |
| 10 | 1.86 | 1.025 | 1.39 | 0.932 | **NL:** Ik zou het vertrouwen in anderen verliezen  **ENG:** I lost trust in others. |
| 11 | 1.72 | 1.020 | 1.39 | 0.992 | **NL:** Ik zou constant boos zijn  **ENG:** I am angry all the time. |
| 12 | 1.51 | 0.954 | 1.21 | 0.925 | **NL:** Ik zou niet de goede persoon zijn die ik nu denk dat ik ben  **ENG:** I am not the good person I thought I was. |
| 13 | 1.74 | 1.093 | 1.33 | 1.001 | **NL:** Ik zou mijn zelf-trots verliezen  **ENG:** I have lost pride in myself. |
| 14 | 1.28 | 0.979 | 1.13 | 0.921 | **NL:** Ik zou niet eerlijk kunnen zijn tegenover andere mensen  **ENG:** I cannot be honest with other people. |

*Note.* In all cases the range was 4, except for item 2 of scenario 2 for which the range was 3.
