# Supplementary material for: Types of decorations, their social meaning and influence on moral injury: A mixed methods approach
Source: PLoS One. 2025 Oct 27;20(10):e0333344. doi: 10.1371/journal.pone.0333344 (PMC12558466; doi:10.1371/journal.pone.0333344)
Supplement: S3 Table — (DOCX) [file pone.0333344.s003.docx]

**S3 Table. Decoration Display Frequencies and Percentages.**

|  |  | Scenario 1 | | | | | Scenario 2 | | | | |
| --- | --- | --- | --- | --- | --- | --- | --- | --- | --- | --- | --- |
|  |  | AccIns | InacIns | AccOut | InacOut | Ot | AccIns | InacIns | AccOut | InacOut | Ot |
| *SEN* | *n* | 133 |  |  |  |  | 118 |  |  |  |  |
|  | *Freq* | 13 | 31 | 25 | 48 | 16 | 6 | 25 | 36 | 40 | 11 |
|  | *Per.* | 9.8 | 23.2 | 18.8 | 36.1 | 12.0 | 5.1 | 21.2 | 30.5 | 33.9 | 9.3 |
| *SER* | *n* | 131 |  |  |  |  | 121 |  |  |  |  |
|  | *Freq.* | 9 | 29 | 35 | 42 | 16 | 12 | 27 | 30 | 40 | 12 |
|  | *Per.* | 6.9 | 21.5 | 25.9 | 31.1 | 11.9 | 9.9 | 22.3 | 24.8 | 33.1 | 9.9 |
| *SYN* | *n* | 122 |  |  |  |  | 121 |  |  |  |  |
|  | *Freq* | 11 | 27 | 27 | 44 | 13 | 12 | 28 | 30 | 31 | 20 |
|  | *Per.* | 9.0 | 22.1 | 22.1 | 36.1 | 10.7 | 9.9 | 23.1 | 24.8 | 25.6 | 16.5 |
| *SYR* | *n* | 121 |  |  |  |  | 121 |  |  |  |  |
|  | *Freq* | 6 | 27 | 24 | 47 | 17 | 11 | 27 | 25 | 46 | 12 |
|  | *Per.* | 5.0 | 22.3 | 19.8 | 38.8 | 14.0 | 9.1 | 22.3 | 20.7 | 38.0 | 9.9 |

*Note.* AccIn = accessible in sight; InacIn = inaccessible in sight; AccOu = accessible out of sight; InacOu = inaccessible out of sight; Ot = Other, namely ….
