# Supplementary material for: Types of decorations, their social meaning and influence on moral injury: A mixed methods approach
Source: PLoS One. 2025 Oct 27;20(10):e0333344. doi: 10.1371/journal.pone.0333344 (PMC12558466; doi:10.1371/journal.pone.0333344)
Supplement: S4 Table — (DOCX) [file pone.0333344.s004.docx]

**S4 Table.** **Event Talking Frequencies and Percentages.**

|  |  | Scenario 1 | | | | | | | | Scenario 2 | | | | | | | |
| --- | --- | --- | --- | --- | --- | --- | --- | --- | --- | --- | --- | --- | --- | --- | --- | --- | --- |
|  |  | Part | CloA | CloN | DFM | Fr | Ac | Str | Col | Part | CloA | CloN | DFM | Fr | Ac | Str | Col |
| *SEN* | *n* | 133 |  |  |  |  |  |  |  | 118 |  |  |  |  |  |  |  |
|  | *Freq* | 102 | 56 | 4 | 9 | 82 | 8 | 8 | 108 | 95 | 62 | 13 | 10 | 69 | 12 | 7 | 100 |
|  | *Per.* | 76.7 | 42.1 | 3.0 | 6.8 | 61.7 | 6.0 | 6.0 | 81.2 | 80.5 | 52.5 | 11.0 | 8.5 | 58.5 | 10.2 | 5.9 | 84.7 |
| *SER* | *n* | 132 |  |  |  |  |  |  |  | 121 |  |  |  |  |  |  |  |
|  | *Freq.* | 106 | 66 | 5 | 9 | 75 | 14 | 13 | 100 | 94 | 63 | 8 | 7 | 63 | 11 | 5 | 99 |
|  | *Per.* | 80.3 | 50.0 | 3.8 | 6.8 | 56.8 | 10.6 | 9.8 | 75.8 | 75.8 | 77.7 | 6.6 | 5.8 | 52.1 | 9.1 | 4.1 | 81.8 |
| *SYN* | *n* | 122 |  |  |  |  |  |  |  | 122 |  |  |  |  |  |  |  |
|  | *Freq* | 96 | 66 | 10 | 9 | 76 | 15 | 11 | 107 | 102 | 58 | 6 | 9 | 68 | 16 | 13 | 99 |
|  | *Per.* | 78.7 | 54.1 | 8.2 | 7.4 | 62.3 | 12.3 | 9.0 | 87.7 | 83.6 | 47.5 | 4.9 | 7.4 | 55.7 | 13.1 | 10.7 | 81.1 |
| *SYR* | *n* | 121 |  |  |  |  |  |  |  | 121 |  |  |  |  |  |  |  |
|  | *Freq* | 107 | 66 | 10 | 10 | 81 | 9 | 4 | 97 | 98 | 63 | 6 | 10 | 83 | 11 | 7 | 98 |
|  | *Per.* | 88.4 | 54.5 | 8.3 | 8.3 | 66.9 | 7.4 | 3.3 | 80.2 | 81.0 | 52.1 | 5.0 | 8.3 | 68.6 | 9.1 | 5.8 | 81.0 |

*Note.* Part = Partner; CloA= close adult family members; CloN = close non-adult family members; DFM = distant family members; Fr = friends; Ac = acquaintances; Str = strangers; Col = colleagues.
