# Supplementary figures and images for: Types of decorations, their social meaning and influence on moral injury: A mixed methods approach
Source: PLoS One. 2025 Oct 27;20(10):e0333344. doi: 10.1371/journal.pone.0333344 (PMC12558466; doi:10.1371/journal.pone.0333344)

**S1 Fig. Scree Plot Exploratory Factor Analysis Scenario 1.**

**
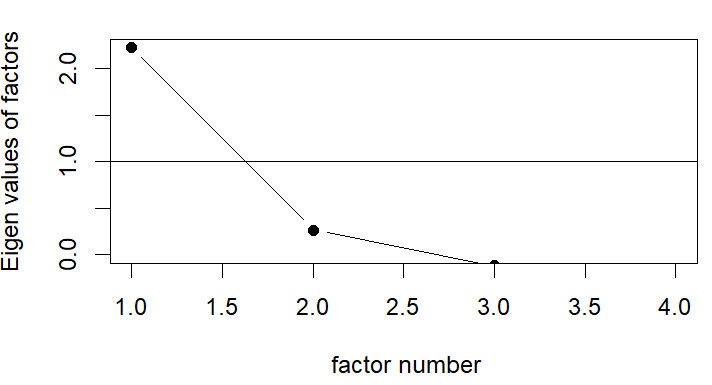
**

Supplement: S1 Fig — (DOCX) [file pone.0333344.s005.docx]

**S2 Fig. Scree Plot Exploratory Factor Analysis Scenario 2.**


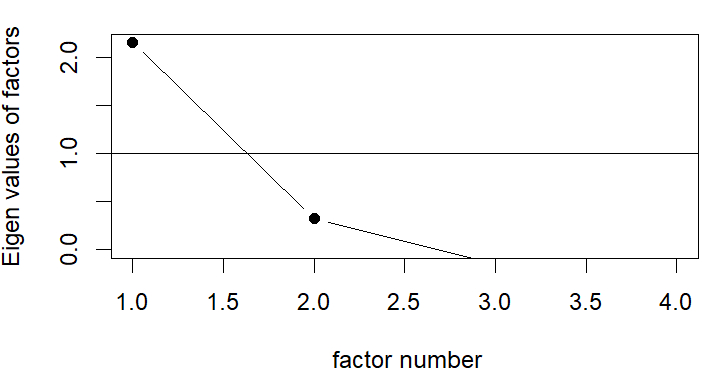

Supplement: S2 Fig — (DOCX) [file pone.0333344.s006.docx]
